# Correction of scaling mismatches in oligonucleotide microarray data

**DOI:** 10.1186/1471-2105-7-251

**Published:** 2006-05-09

**Authors:** Martino Barenco, Jaroslav Stark, Daniel Brewer, Daniela Tomescu, Robin Callard, Michael Hubank

**Affiliations:** 1 lnstitute of Child Health, University College London, UK; 2 CoMPLEX, University College London, UK; 3 Department of Mathematics, Imperial College London, UK

## Abstract

**Background:**

Gene expression microarray data is notoriously subject to high signal variability. Moreover, unavoidable variation in the concentration of transcripts applied to microarrays may result in poor scaling of the summarized data which can hamper analytical interpretations. This is especially relevant in a systems biology context, where systematic biases in the signals of particular genes can have severe effects on subsequent analyses. Conventionally it would be necessary to replace the mismatched arrays, but individual time points cannot be rerun and inserted because of experimental variability. It would therefore be necessary to repeat the whole time series experiment, which is both impractical and expensive.

**Results:**

We explain how scaling mismatches occur in data summarized by the popular MAS5 (GCOS; Affymetrix) algorithm, and propose a simple recursive algorithm to correct them. Its principle is to identify a set of constant genes and to use this set to rescale the microarray signals. We study the properties of the algorithm using artificially generated data and apply it to experimental data. We show that the set of constant genes it generates can be used to rescale data from other experiments, provided that the underlying system is similar to the original. We also demonstrate, using a simple example, that the method can successfully correct existing imbalancesin the data.

**Conclusion:**

The set of constant genes obtained for a given experiment can be applied to other experiments, provided the systems studied are sufficiently similar. This type of rescaling is especially relevant in systems biology applications using microarray data.

## Background

Gene expression profiling using microarrays has become a popular technique in modern biochemical research. One of the commonest microarray platforms in use is the high-density oligonucleotide array introduced by Affymetrix (Santa Clara, CA). In the Affymetrix system, biotinylated cRNA generated from the sample of interest is hybridised to the array and detected using fluorescently-labelled streptavidin. A number of different expression summary algorithms are available to derive the concentration of each transcript from the intensity of fluorescence. These include MAS5 (Affymetrix) [1], RMA [2], MBEI [3,4].

It is important to use the most accurate and precise methods for calculating gene expression levels from microarray data. MAS5, RMA and MBEI offer different solutions to this problem. On Affymetrix arrays, transcripts are represented by multiple (typically 11) pairs of 25-mer oligonucleotides termed a probeset. One of each pair is a perfect match (PM) for the target and the other is a mismatch control (MM). In MAS5, signal values from MM oligonucleotides are subtracted from PM oligonucleotide signals (if the MM value is greater than the PM value, an "ideal mismatch" (IM) value is subtracted from the PM value). RMA and MBEI use only the perfect match signal. In MAS5 there is no attempt to correct non-linearities in the signal intensity response resulting for example from different overall transcript concentrations. In other words, MAS5 treats microarrays individually in contrast to RMA and MBEI, which apply a ranking-type algorithm to arrive at a signal value, and require multiple microarrays. However, it is important to note that the nomalization steps applied at the probe level in both MBEI and RMA even out non-linearities across microarray experiments, but do not correct them. The other distinctive characteristic of MAS5 is that anaysis of spike-in data has shown it to be more accurate than the other methods cited [5,6]. This higher accuracy, however, comes at the cost of a lower precision -or higher variablity- at low expression values. These differences in method result in differences in performance and a trade-off between accuracy and precision. Researchers select their preferred expression summary algorithm depending on their particular experimental application.

To date, expression profiling has been largely limited to applications in class discovery or prediction (geared towards molecular diagnostics), and in relatively simple functional studies involving direct comparisons of microarrays. Increasingly however, there is a move towards the mathematical modelling of dynamic gene networks. These systems biology applications typically require a sequence of microarray measurements in time which are applied in conjunction with dynamical models. In this context, measurement accuracy is paramount otherwise it would be impossible to arrive at accurate solutions to kinetic parameter estimates, such as transcript degradation rates. Therefore in systems biology, the accuracy of the measurements takes precedence over other considerations such as their precision. The higher accuracy achieved by MAS5 makes it the method of choice for gene network modelling.

Systems biology applications also raise practical issues. Time course data collection is demanding on resources as replicate RNA samples must be isolated for each timepoint. Because of experimental variability, individual timepoints cannot be rerun separately. Rejection of data from outlier arrays in a time course experiment should therefore entail the replacement of the whole time course. In complex experimental systems this is often unfeasible on the grounds of both practicality and cost. Consequently there is a demand for methods for renormalising, and thus retaining, sub-optimal arrays. These methods would benefit all microarray applications, but would be of particular importance where the data is applied in modeling contexts. In this paper we therefore concentrate on renormalisation of MAS5 generated data to more accurately reflect expression values.

In MAS5, in order to make microarrays comparable, the trimmed average of all signals on a microarray is set to some predefined value. Unlike other methods [2,7], this simple scaling does not attempt to even out distortions in signal response (See Figure 1A). A knock-on effect is that, because the Affymetrix scaling procedure takes into account almost all the signals on the microarrays, those genes lying in the median range have a scaling mismatch (See Figure 1A first panel). Data imbalances that result become particularly visible in time course applications (Figure 1B). In this paper, we propose a procedure that corrects these scaling mismatches. We do not aim to correct for distortions occurring across the whole signal range but we rather try to concentrate on those genes whose intensity is in the median signal range because they are particularly important in modelling situations. It is also in this range that scaling mismatches can have have the most deleterious effects because they are commensurate -if not bigger- than the measurement error (at low expression level, measurement errors swamp these scaling mismatches). The problem of signal *saturation *affecting strongly expressed genes has been identified and partially corrected elsewhere [8,9]. In this paper, and for notational convenience only, we will use the term "gene" in place of "probe set", even though a given gene can have several probe sets.

## Results and discussion

Our method supposes there exists a set of constant genes, i.e. genes whose expression levels are unaffected under the experimental conditions, and that these genes can be used to accurately normalize the data. This idea of using constant genes has already been proposed [10], it was however in the context of two-colour cDNA microarrays. Our method consists of identifying this set of constant genes in an iterative fashion. In a first step a set of constant genes is singled out on the basis of some dispersion criteria, we call this set the initial set. The genes belonging to this set are used to normalize the microarrays. In a subsequent step, a new set of unchanging genes is defined using the same criteria. Several such steps are performed in sequence until the set of constant genes is unchanged from one step to the next. At this point the method is said to have converged and the corresponding set of constant probe sets is used to rescale the whole experiment (for a thorough description see Methods section).

To study the properties of the method, we generated artificial data. This allowed us to explore its convergence, increase its efficiency and define criteria for detecting hypothetical (and unlikely) situations in which a set of constant genes is non-existent. The results of these purely in silico explorations are described in the the first part of this section, the description of the setup is deferred to the methods section. In the second part, we apply the algorithm to real experimental data and show that it converges to a unique set of genes and that the set of constant genes can be re-used with a different microarray dataset, provided that the tissues involved are sufficiently similar. We demonstrate, using a simple time course interpolation example, that the resulting rescaling does indeed correct imbalances in the data and compare these results with another popular normalization procedure (quantile normalization) and with RMA-summarized data.

### In Silico explorations

Artificial data was generated for 10'000 genes on 7 microarrays, a small portion (5%) of these genes was set to be constant. The scaling of these data was then perturbed with varying severity and for each perturbation our rescaling algorithm was applied. This setup is thoroughly described in the Methods section.

Each individual run was assigned to one of the three following groups:

• *Good *runs were those for which the size of the final set exceeded 100.

• In contrast, *poor *runs were those for which the size of the final was less than or equal to 100.

• In the special case where the algorithm failed to find a big enough (> 2) initial set, three genes known to be constant were forced into the initial set (recall that 5% of the genes were set to be constant). We call these *helped *runs.

For each run, we computed an accuracy index (see methods) indicating whether the artificially induced perturbations were accurately corrected. This index can take non-negative values and must be as close to zero as possible. We also monitored the speed of convergence, i.e. the number of iterations steps the algorithm takes before convergence.

#### Artificial data: results

From this first series of runs we can conclude that the amount of perturbation affects the speed of convergence, the bigger the perturbation, the longer it takes for the algorithm to converge (Figure 2, top plot). The number of steps does not exceed 20, which makes the algorithm extremely fast (< 1 minute on a standard PC). Secondly, the threshold of 100 used to separate good and poor runs correctly identifies those runs that yield a good accuracy (Figure 2, median plot). All good and helped runs, with a final set size far higher than our set threshold of 100 have a very good accuracy (< 1%) whereas poor runs, with a much smaller set size are not accurate. Note also that the threshold of 100 could have been safely set anywhere between 50 and 350 (Figure 2, bottom plot).

Helped runs constitute a special case. Despite starting with a high perturbation – which is why they need help in the first place – they end up being very similar to good runs with both a good accuracy and a large final set. The inclusion of genes known to be constant accelerates convergence so that helped runs converge within 5–7 iterations. Helped runs occur mainly when the perturbation is large. This demonstrates that feeding some known constant genes into the initial iteration is a good way to "seed" the algorithm and has the added bonus of speeding up the (already quick) process. With real data, standard housekeeping genes (Affymetrix identifier ranging from 20000_*s*_*at *to 20099_*s*_*at *on the U133 Array) can fulfill that role.

Although they might not end up in the final set, our experience is that the inclusion of some of these in the initial set does indeed set the algorithm onto the right path.

Poor runs were defined by setting a limit on the size of the final set. For these cases, it is easy to verify that the final set, on top of being small, has a small "basin of attraction". We perturbed the initial guess by removing and adding genes. This produced *different *final sets thereby confirming the poor quality of these runs. In contrast, good and helped runsproduced stable final sets (data not shown). Hence, poor quality is fairly easy to detect and can be remedied by supplying the algorithm with a small set of genes that are suspected of being constant, i.e. to create a "helped" run.

In the good and helped runs, the final set of constant genes contains around 400 genes. Hence, not all constant genes (recall that 500 of those were generated) are included in this final set. A less severe selection criterion would select more constant genes. The final set also includes a small number of genes (< 10) that were not expected to be constant. But, as the accuracy plot testifies, their presence does not have an adverse effect on the final result. With real data, the boundary between constant and non-constant genes is blurry, and lightly downregulated genes are likely to be balanced by lightly upregulated ones. This hypothesis is also at the heart of the centralization algorithm presented in [11].

From this first series of simulations, we conclude that the quality of a run can simply be assessed by the size of the final set and that a good way to ensure fast and accurate convergence is to feed the starting set with genes known to be constant. In a real situation, standard housekeeping genes can be used to that effect.

#### Algorithm performance in the absence of constant genes

In the controlled experiments described above, we set a small proportion of genes to be constant. To further test the main features of the algorithm we ran it in the absence of constant genes, the results are shown in Figure 3. All the runs are flagged as poor because of the small size of the final sets. The diversity of these runs is reflected in the variety of both the size of the final sets and the measured accuracies. The latter are all above 10%. Thus, the algorithm fares badly in the absence of constant genes but this absence is easily deduced from the lack of a large strongly attracting final set. With the real experimental data presented below, this does not happen, and we believe that in most real situations there should be a sufficiently large subset of constant genes.

In summary, we recommend including prior information, by supplying the algorithm with a set of genes known or likely to be constant. A strong sign that the algorithm has converged correctly is that the resulting final set is large, unique and stable, i.e. immune to the inclusion of random genes and/or the deletions of genes belonging to it. Finally, if these properties of the final set are not met, then this suggests that there are no constant genes. In our experience, this is unlikely to happen in reality.

### Experimental data

Cells from the human T-cell line (MOLT4) were submitted to ionising radiation of 5 Gy using a ^137^*Cs *source. RNA was prepared and labelled using standard procedures. Affymetrix U133A microarrays were then run on biological triplicates of transcripts taken 2,4,6,8,10 and 12 hours after irradiation. Triplicates were also taken at 0 hours, i.e. just before irradiation. These 21 microarrays vary in average signal intensity, by this we mean that some are "darker" than others, indicating a lower transcript concentration. We will refer to this data set as the 5Gy experiment. Another set of chips were run under different conditions, the irradiation dose was smaller (1Gy) than in the previous time series (5Gy). Biological triplicates were taken 4, 8 and 12 hours after irradiation, the 0 hours microarrays were the same as in the 5Gy experiment, therefore there is a slight overlap between the two experiments. This dataset, consisting of 12 microarrays, will be referred to as the 1Gy experiment. The data was summarized with MAS5 using standard parameters.

#### 5Gy experiment

We first applied the algorithm to the 5Gy experiment. As a measure of the variability of a gene, we used the second formula (5) given in the methods section and fixed the variability limit to 0.2 to match our estimation of the relative error. This condition was applied to each replicate separately (if a replicate failed, then the gene would be excluded from the constant set) rather than globally on the 21 microarrays.

Further conditions (denoted in the methods section equation (6) by *C*(*i, A*))were as follows:

• To avoid including genes with a low expression and absent genes an upper limit of 0.001 was set on the detection *p*-value provided by MAS5.

• An upper limit on the signal value of the 0 hours microarray of the first replicate was set to 2000.

This limit empirically corresponds to the point where distortions in the upper range of the signal become apparent (see Figure 1).

The first replicate of the 0 hours microarray was set to be the reference microarray. The algorithm converged in six steps to an invariant final set composed of 1194 genes out of a total of 1675 genes that passed the 0.001 p-value and 2000 intensity filtering. The resulting rescaling factors are given in Table 1.

To test the stability of the final set, several further runs were performed using perturbed versions of the original data. To do this, we chose initial values of pa0
 MathType@MTEF@5@5@+=feaafiart1ev1aaatCvAUfKttLearuWrP9MDH5MBPbIqV92AaeXatLxBI9gBaebbnrfifHhDYfgasaacH8akY=wiFfYdH8Gipec8Eeeu0xXdbba9frFj0=OqFfea0dXdd9vqai=hGuQ8kuc9pgc9s8qqaq=dirpe0xb9q8qiLsFr0=vr0=vr0dc8meaabaqaciaacaGaaeqabaqabeGadaaakeaacqWGWbaCdaqhaaWcbaGaemyyaegabaGaeGimaadaaaaa@307B@ not equal to 1 (pa0
 MathType@MTEF@5@5@+=feaafiart1ev1aaatCvAUfKttLearuWrP9MDH5MBPbIqV92AaeXatLxBI9gBaebbnrfifHhDYfgasaacH8akY=wiFfYdH8Gipec8Eeeu0xXdbba9frFj0=OqFfea0dXdd9vqai=hGuQ8kuc9pgc9s8qqaq=dirpe0xb9q8qiLsFr0=vr0=vr0dc8meaabaqaciaacaGaaeqabaqabeGadaaakeaacqWGWbaCdaqhaaWcbaGaemyyaegabaGaeGimaadaaaaa@307B@ is the initial perturbation coefficient, see methods). For example, pa0
 MathType@MTEF@5@5@+=feaafiart1ev1aaatCvAUfKttLearuWrP9MDH5MBPbIqV92AaeXatLxBI9gBaebbnrfifHhDYfgasaacH8akY=wiFfYdH8Gipec8Eeeu0xXdbba9frFj0=OqFfea0dXdd9vqai=hGuQ8kuc9pgc9s8qqaq=dirpe0xb9q8qiLsFr0=vr0=vr0dc8meaabaqaciaacaGaaeqabaqabeGadaaakeaacqWGWbaCdaqhaaWcbaGaemyyaegabaGaeGimaadaaaaa@307B@ = 1 + *ε*, where *ε *is a random variable uniformly distributed on (-1/2,1/2). In all of these additional runs the algorithm converged to a very similar final set (the overlap was > 99% in each case), which confirms its robustness of the original set.

Unsurprisingly, it is the darkest microarrays that require the most intensive rescaling (replicate 2: 8 hours/replicate 3: 8 and 10 hours): Table 2 below shows the original scaling factors applied by MAS5. However this correlation breaks down for lower scaling factors. It is important to stress that our rescaling factors do not replace MAS5's original scaling factors but complement them. For example, the total scaling applied to the raw data obtained from the microarray run at eight hours (sample 2) is 3.010 * 1.22.

As intended, the expression levels of the genes composing the constant set span a smaller range than the overall expression level measured on a microarray (Figure 4). Note that the majority of gene with signals below 20 are considered absent by the MAS5 algorithm.

#### 1Gy experiment

Our rescaling algorithm was run with the 1Gy experiment data set under the same conditions as in the 5Gy experiment, resulting in a set of 1568 constant genes. In this 1Gy experiment, 2944 genes in total passed the 0.001 p-value and 2000 signal intensity filtering. The corresponding rescaling factors are shown in Table 3 below.

With respect to the 5Gy experiment, the quality of the microarrays used in this second experiment is more uniform, which results in rescaling factors that are closer to 1. Notice as well that the number of genes included in the constant set is larger: 1568 instead of 1194. This is also because in the 1Gy experiment there were less microarrays of lower quality, causing more genes in this experiment to pass the filtering step.

#### Lists of constant genes are re-useable

Of the 1568 genes flagged as constant in the 1Gy experiment, 826 are also in the the list of constant genes for the 5Gy experiment (hence 742 genes are included in the 1Gy list only and 368 in the 5Gy list only). Despite this incomplete overlap, we show below that the usage of such lists does not have to be restricted to the experiments from which they were extracted.

We found that calculating rescaling factors for the 1Gy data set using the list of 1194 genes identified though the 5Gy experiment produced values very similar to those obtained with the 1Gy data alone (Figure 5, right). The biggest absolute difference between the two sets of values occurs for the first sample microarray 4 hours after irradiation and is equal to 0.013 (the average of these absolute differences across all microarrays is equal to 0.005).

The same conclusion can be reached when the experiment roles are swapped. The rescaling factors for the 5Gy data obtained using the 1Gy set of constant genes are compared with the original values in Figure 5 (left plot) and the biggest absolute difference is 0.024 for the third replicate 2 hours after irradiation, the average of these absolute differences being equal to 0.007.

Therefore, provided that the experimental conditions are not too dissimilar (same cell line and treatment), we conclude that the list of constant genes obtained through a given experiment can be safely used to calibrate data obtained from another experiment.

#### Why is rescaling necessary?

The rescaling factors shown in Table 1 might seem small with respect to the measurement error that prevails in microarray experiments and they may have little impact in simple screening or comparison-type analyses. However, increasing numbers of microarray experiments are carried out in a systems biology context (see [12] for a short review), in which proper scaling of the data is crucial to obtain sound interpretations.

To illustrate this point, we performed a simple time course analysis. Intermediate points (at 2,4,6,8 and 10 hours) were "predicted" using a linear interpolation procedure on neighbouring time points, for each replicate and gene (Figure 6). Each plot shows the quartiles of relative interpolation error ((true value -interpolated value)/true value) for the first and the second replicate, before (Figure 6A) and after rescaling (Figure 6B). Non-rescaled data is severely skewed, this is especially the case for those time points suffering from ascaling mismatch such as the one illustrated in figure 1 (replicate 2, 8 hours). Rescaling the data corrects for this mismatch: the interpolation errors are much more centered in the rescaled situation. We have used this rescaled data in a modeling context to predict targets of p53, an important transcription factor involved in the cell response to DNA damage [13].

We also compared our method to another popular normalization method: quantile normalization [14]. This normalization step is the one used in the RMA summary method, albeit at the probe level. We applied it instead to MAS5 summarized values and the results are shown in figure 6C. As can be readily seen from this example, quantile normalization fails to accurately correct for scaling imbalances in the data. Similarly, we performed the same interpolation analysis on RMA-summarized probe-level data (figure 6D). RMA does not perform well in this situation, we believe this is because the lesser quality microarrays have a deleterious effect on the whole dataset.

## Conclusion

Inaccurate scaling of median range signal values can have significant deleterious effects on mathematical modelling of microarray data. Experimental correction of problematic arrays can be expensive and impractical. We have therefore developed a method for rescaling arrays to avoid the unnecessary loss of otherwise valid data. We present an algorithm designed to identify a set of constant genes in oligonucleotide microarray experiments. Applying this algorithm to artificially generated data allowed us to identify the conditions under which this set can be used for the correction of scaling mismatches. We applied the algorithm successfully to real experimental data and showed that the set of constant genes obtained for a given experiment can be generally applied, provided the systems studied are sufficiently similar. This type of rescaling is especially relevant, if not crucial, in modelling applications of microarray data.

## Methods

### Description of the algorithm

Let *x*_*i*,*a *_be the signal for gene *i *as it is measured in microarray *a*. We hypothesise that this value can be decomposed as follows:

*x*_*i*,*a *_= *k*_*a*_*s*_*i*,*a *_exp(*ε*),     (1)

where *s*_*i*,*a *_is the "true" signal for gene *i *in microarray *a *(the value of the signal depends on the microarray as well, but for purely biological reasons). The third term *exp*(*ε*) is a multiplicative noise term, *ε *is a random variable identically distributed across all genes and microarrays with mean zero, and standard deviation typically around 0.15 – 0.20 which matches the average relative error (of 15% – 20%) generally observed in microarray data. The focus here is on the first component *k*_*a*_, which results from inaccurate normalisation. In other words, one wishes to find a rescaling factor *p*_*a *_such that

*p*_*a*_*k*_*a *_= 1.

Note that there is no "true" level for the signal, so that a more accurate formulation would be that one wishes to find *p*_*a*_such that *p*_*a*_*k*_*a *_= *C *for all microarrays a, where *C *is some user-defined constant. However, we will define a particular microarray *r *to be the reference *(k*_*r *_= 1) in order to retain the above formulation.

The proposed algorithm to determine the *p*_*a*_'s is simple, it assumes that there exists a set of genes *U *whose expression is unchanged across all microarrays:

*U *= {*i *: *s*_*i*,*a *_= *s*_*i *_> 0 ∀ *a*}.

If *U *is known, an accurate estimate of *p*_*a *_can be obtained via a ratio of geometric averages for the relevant microarrays:

p˜a=(∏i∈Uxi,r)1/#(U)(∏i∈Uxi,a)1/#(U),     (2)
MathType@MTEF@5@5@+=feaafiart1ev1aaatCvAUfKttLearuWrP9MDH5MBPbIqV92AaeXatLxBI9gBaebbnrfifHhDYfgasaacH8akY=wiFfYdH8Gipec8Eeeu0xXdbba9frFj0=OqFfea0dXdd9vqai=hGuQ8kuc9pgc9s8qqaq=dirpe0xb9q8qiLsFr0=vr0=vr0dc8meaabaqaciaacaGaaeqabaqabeGadaaakeaacuWGWbaCgaacamaaBaaaleaacqWGHbqyaeqaaOGaeyypa0ZaaSaaaeaadaqadaqaamaarababaGaemiEaG3aaSbaaSqaaiabdMgaPjabcYcaSiabdkhaYbqabaaabaGaemyAaKMaeyicI4SaemyvaufabeqdcqGHpis1aaGccaGLOaGaayzkaaWaaWbaaSqabeaacqaIXaqmcqGGVaWlcqGGJaWidaqadaqaaiabdwfavbGaayjkaiaawMcaaaaaaOqaamaabmaabaWaaebeaeaacqWG4baEdaWgaaWcbaGaemyAaKMaeiilaWIaemyyaegabeaaaeaacqWGPbqAcqGHiiIZcqWGvbqvaeqaniabg+GivdaakiaawIcacaGLPaaadaahaaWcbeqaaiabigdaXiabc+caViabcocaJmaabmaabaGaemyvaufacaGLOaGaayzkaaaaaaaakiabcYcaSiaaxMaacaWLjaWaaeWaaeaacqaIYaGmaiaawIcacaGLPaaaaaa@59D8@

where *#(U) *denotes the number of elements in the set *U*. We show below that p˜a
 MathType@MTEF@5@5@+=feaafiart1ev1aaatCvAUfKttLearuWrP9MDH5MBPbIqV92AaeXatLxBI9gBaebbnrfifHhDYfgasaacH8akY=wiFfYdH8Gipec8Eeeu0xXdbba9frFj0=OqFfea0dXdd9vqai=hGuQ8kuc9pgc9s8qqaq=dirpe0xb9q8qiLsFr0=vr0=vr0dc8meaabaqaciaacaGaaeqabaqabeGadaaakeaacuWGWbaCgaacamaaBaaaleaacqWGHbqyaeqaaaaa@2F9B@ is a good approximation for *p*_*a*_:

p˜a
 MathType@MTEF@5@5@+=feaafiart1ev1aaatCvAUfKttLearuWrP9MDH5MBPbIqV92AaeXatLxBI9gBaebbnrfifHhDYfgasaacH8akY=wiFfYdH8Gipec8Eeeu0xXdbba9frFj0=OqFfea0dXdd9vqai=hGuQ8kuc9pgc9s8qqaq=dirpe0xb9q8qiLsFr0=vr0=vr0dc8meaabaqaciaacaGaaeqabaqabeGadaaakeaacuWGWbaCgaacamaaBaaaleaacqWGHbqyaeqaaaaa@2F9B@ ≈ *p*_*a*_.     (3)

Our aim is to determine *U*. To do this, we first determine a set of genes *U*_1 _which have a low "variability" V
 MathType@MTEF@5@5@+=feaafiart1ev1aaatCvAUfKttLearuWrP9MDH5MBPbIqV92AaeXatLxBI9gBamrtHrhAL1wy0L2yHvtyaeHbnfgDOvwBHrxAJfwnaebbnrfifHhDYfgasaacH8akY=wiFfYdH8Gipec8Eeeu0xXdbba9frFj0=OqFfea0dXdd9vqai=hGuQ8kuc9pgc9s8qqaq=dirpe0xb9q8qiLsFr0=vr0=vr0dc8meaabaqaciaacaGaaeqabaWaaeGaeaaakeaaimaacqWFveVvaaa@384B@. As a measure of variability, we could use the coefficient of variation, which is given by

V(i,A)=x¯i−1(1n−1∑a=1n(xi,a−x¯i)2)1/2     (4)
 MathType@MTEF@5@5@+=feaafiart1ev1aaatCvAUfKttLearuWrP9MDH5MBPbIqV92AaeXatLxBI9gBamrtHrhAL1wy0L2yHvtyaeHbnfgDOvwBHrxAJfwnaebbnrfifHhDYfgasaacH8akY=wiFfYdH8Gipec8Eeeu0xXdbba9frFj0=OqFfea0dXdd9vqai=hGuQ8kuc9pgc9s8qqaq=dirpe0xb9q8qiLsFr0=vr0=vr0dc8meaabaqaciaacaGaaeqabaWaaeGaeaaakeaaimaacqWFveVvdaqadaqaaiabdMgaPjabcYcaSiabdgeabbGaayjkaiaawMcaaiabg2da9iqbdIha4zaaraWaa0baaSqaaiabdMgaPbqaaiabgkHiTiabigdaXaaakmaabmaabaWaaSaaaeaacqaIXaqmaeaacqWGUbGBcqGHsislcqaIXaqmaaWaaabCaeaadaqadaqaaiabdIha4naaBaaaleaacqWGPbqAcqGGSaalcqWGHbqyaeqaaOGaeyOeI0IafmiEaGNbaebadaWgaaWcbaGaemyAaKgabeaaaOGaayjkaiaawMcaamaaCaaaleqabaGaeGOmaidaaaqaaiabdggaHjabg2da9iabigdaXaqaaiabd6gaUbqdcqGHris5aaGccaGLOaGaayzkaaWaaWbaaSqabeaacqaIXaqmcqGGVaWlcqaIYaGmaaGccaWLjaGaaCzcamaabmaabaGaeGinaqdacaGLOaGaayzkaaaaaa@6280@

where *A *is the set *A *= {1, ..., *n*} (the set of microarrays), *x*_*i*,*a*_, *a *= 1, ..., *n *denotes *n *observations and x¯i
 MathType@MTEF@5@5@+=feaafiart1ev1aaatCvAUfKttLearuWrP9MDH5MBPbIqV92AaeXatLxBI9gBaebbnrfifHhDYfgasaacH8akY=wiFfYdH8Gipec8Eeeu0xXdbba9frFj0=OqFfea0dXdd9vqai=hGuQ8kuc9pgc9s8qqaq=dirpe0xb9q8qiLsFr0=vr0=vr0dc8meaabaqaciaacaGaaeqabaqabeGadaaakeaacuWG4baEgaqeamaaBaaaleaacqWGPbqAaeqaaaaa@2FC4@ is the arithmetic mean of the *x*_*i*,*a *_across all *a*'s. We thus average the readings for a particular gene across several microarrays. However, because of the multiplicative context, it is preferable to use a geometric average, so that

V(i,A)=(∏a=1nMax(xi,ax¯ig,x¯igxi,a))1n−1−1,     (5)
 MathType@MTEF@5@5@+=feaafiart1ev1aaatCvAUfKttLearuWrP9MDH5MBPbIqV92AaeXatLxBI9gBamrtHrhAL1wy0L2yHvtyaeHbnfgDOvwBHrxAJfwnaebbnrfifHhDYfgasaacH8akY=wiFfYdH8Gipec8Eeeu0xXdbba9frFj0=OqFfea0dXdd9vqai=hGuQ8kuc9pgc9s8qqaq=dirpe0xb9q8qiLsFr0=vr0=vr0dc8meaabaqaciaacaGaaeqabaWaaeGaeaaakeaaimaacqWFveVvdaqadaqaaiabdMgaPjabcYcaSiabdgeabbGaayjkaiaawMcaaiabg2da9maabmaabaWaaebCaeaacqWGnbqtcqWGHbqycqWG4baEdaqadaqaamaalaaabaGaemiEaG3aaSbaaSqaaiabdMgaPjabcYcaSiabdggaHbqabaaakeaacuWG4baEgaqeamaaDaaaleaacqWGPbqAaeaacqWGNbWzaaaaaOGaeiilaWYaaSaaaeaacuWG4baEgaqeamaaDaaaleaacqWGPbqAaeaacqWGNbWzaaaakeaacqWG4baEdaWgaaWcbaGaemyAaKMaeiilaWIaemyyaegabeaaaaaakiaawIcacaGLPaaaaSqaaiabdggaHjabg2da9iabigdaXaqaaiabd6gaUbqdcqGHpis1aaGccaGLOaGaayzkaaWaaWbaaSqabeaadaWcbaadbaGaeGymaedabaGaemOBa4MaeyOeI0IaeGymaedaaaaakiabgkHiTiabigdaXiabcYcaSiaaxMaacaWLjaWaaeWaaeaacqaI1aqnaiaawIcacaGLPaaaaaa@6B60@

where x¯ig
 MathType@MTEF@5@5@+=feaafiart1ev1aaatCvAUfKttLearuWrP9MDH5MBPbIqV92AaeXatLxBI9gBaebbnrfifHhDYfgasaacH8akY=wiFfYdH8Gipec8Eeeu0xXdbba9frFj0=OqFfea0dXdd9vqai=hGuQ8kuc9pgc9s8qqaq=dirpe0xb9q8qiLsFr0=vr0=vr0dc8meaabaqaciaacaGaaeqabaqabeGadaaakeaacuWG4baEgaqeamaaDaaaleaacqWGPbqAaeaacqWGNbWzaaaaaa@311C@ is the geometric mean. We therefore define

*U*_1 _= {*i *: V
 MathType@MTEF@5@5@+=feaafiart1ev1aaatCvAUfKttLearuWrP9MDH5MBPbIqV92AaeXatLxBI9gBamrtHrhAL1wy0L2yHvtyaeHbnfgDOvwBHrxAJfwnaebbnrfifHhDYfgasaacH8akY=wiFfYdH8Gipec8Eeeu0xXdbba9frFj0=OqFfea0dXdd9vqai=hGuQ8kuc9pgc9s8qqaq=dirpe0xb9q8qiLsFr0=vr0=vr0dc8meaabaqaciaacaGaaeqabaWaaeGaeaaakeaaimaacqWFveVvaaa@384B@(*i, A*) <v_* _C
 MathType@MTEF@5@5@+=feaafiart1ev1aaatCvAUfKttLearuWrP9MDH5MBPbIqV92AaeXatLxBI9gBamrtHrhAL1wy0L2yHvtyaeHbnfgDOvwBHrxAJfwnaebbnrfifHhDYfgasaacH8akY=wiFfYdH8Gipec8Eeeu0xXdbba9frFj0=OqFfea0dXdd9vqai=hGuQ8kuc9pgc9s8qqaq=dirpe0xb9q8qiLsFr0=vr0=vr0dc8meaabaqaciaacaGaaeqabaWaaeGaeaaakeaaimaacqWFce=qaaa@3825@ (*i, A*)},

where *A *is the set of microarrays and v_* _is a parameter. C
 MathType@MTEF@5@5@+=feaafiart1ev1aaatCvAUfKttLearuWrP9MDH5MBPbIqV92AaeXatLxBI9gBamrtHrhAL1wy0L2yHvtyaeHbnfgDOvwBHrxAJfwnaebbnrfifHhDYfgasaacH8akY=wiFfYdH8Gipec8Eeeu0xXdbba9frFj0=OqFfea0dXdd9vqai=hGuQ8kuc9pgc9s8qqaq=dirpe0xb9q8qiLsFr0=vr0=vr0dc8meaabaqaciaacaGaaeqabaWaaeGaeaaakeaaimaacqWFce=qaaa@3825@ (*i, A*) is a boolean variable that denotes another condition (or set of conditions) on the *i*th gene and is not of direct concern here. For example, via C
 MathType@MTEF@5@5@+=feaafiart1ev1aaatCvAUfKttLearuWrP9MDH5MBPbIqV92AaeXatLxBI9gBamrtHrhAL1wy0L2yHvtyaeHbnfgDOvwBHrxAJfwnaebbnrfifHhDYfgasaacH8akY=wiFfYdH8Gipec8Eeeu0xXdbba9frFj0=OqFfea0dXdd9vqai=hGuQ8kuc9pgc9s8qqaq=dirpe0xb9q8qiLsFr0=vr0=vr0dc8meaabaqaciaacaGaaeqabaWaaeGaeaaakeaaimaacqWFce=qaaa@3825@ (*i, A*), we might try and avoid including in the set of constant genes those genes that are flagged absent by MAS5 throughout the experiment.

We then assume *U*_1 _is *U *and we use (2) to compute estimated rescaling factors p˜a
 MathType@MTEF@5@5@+=feaafiart1ev1aaatCvAUfKttLearuWrP9MDH5MBPbIqV92AaeXatLxBI9gBaebbnrfifHhDYfgasaacH8akY=wiFfYdH8Gipec8Eeeu0xXdbba9frFj0=OqFfea0dXdd9vqai=hGuQ8kuc9pgc9s8qqaq=dirpe0xb9q8qiLsFr0=vr0=vr0dc8meaabaqaciaacaGaaeqabaqabeGadaaakeaacuWGWbaCgaacamaaBaaaleaacqWGHbqyaeqaaaaa@2F9B@. The *x*_*i*,*a*_'s can then be updated, but this is likely to modify the composition of the set constant genes so that we might have to perform a similar step again, therefore the set *U *has to be determined in an *iterative *fashion. The full description of the *k*th step is

1. Determine a putative set of "constant" genes using

*U*_*k *_= {*i *:  (*i, A, k *- 1) <v*, C
 MathType@MTEF@5@5@+=feaafiart1ev1aaatCvAUfKttLearuWrP9MDH5MBPbIqV92AaeXatLxBI9gBamrtHrhAL1wy0L2yHvtyaeHbnfgDOvwBHrxAJfwnaebbnrfifHhDYfgasaacH8akY=wiFfYdH8Gipec8Eeeu0xXdbba9frFj0=OqFfea0dXdd9vqai=hGuQ8kuc9pgc9s8qqaq=dirpe0xb9q8qiLsFr0=vr0=vr0dc8meaabaqaciaacaGaaeqabaWaaeGaeaaakeaaimaacqWFce=qaaa@3825@ (*i, A*)}.     (6)

In the equation above, a parameter *k *- 1 has been added to the function V
 MathType@MTEF@5@5@+=feaafiart1ev1aaatCvAUfKttLearuWrP9MDH5MBPbIqV92AaeXatLxBI9gBamrtHrhAL1wy0L2yHvtyaeHbnfgDOvwBHrxAJfwnaebbnrfifHhDYfgasaacH8akY=wiFfYdH8Gipec8Eeeu0xXdbba9frFj0=OqFfea0dXdd9vqai=hGuQ8kuc9pgc9s8qqaq=dirpe0xb9q8qiLsFr0=vr0=vr0dc8meaabaqaciaacaGaaeqabaWaaeGaeaaakeaaimaacqWFveVvaaa@384B@, to underline the fact that the coefficient of variation has to be determined with updated values of the signal.

2. Update the rescaling factors using

pak=pak−1(∏i∈Ukxi,rk−1xi,ak−1)1/#(Uk),
 MathType@MTEF@5@5@+=feaafiart1ev1aaatCvAUfKttLearuWrP9MDH5MBPbIqV92AaeXatLxBI9gBaebbnrfifHhDYfgasaacH8akY=wiFfYdH8Gipec8Eeeu0xXdbba9frFj0=OqFfea0dXdd9vqai=hGuQ8kuc9pgc9s8qqaq=dirpe0xb9q8qiLsFr0=vr0=vr0dc8meaabaqaciaacaGaaeqabaqabeGadaaakeaacqWGWbaCdaqhaaWcbaGaemyyaegabaGaem4AaSgaaOGaeyypa0JaemiCaa3aa0baaSqaaiabdggaHbqaaiabdUgaRjabgkHiTiabigdaXaaakmaabmaabaWaaebuaeaadaWcaaqaaiabdIha4naaDaaaleaacqWGPbqAcqGGSaalcqWGYbGCaeaacqWGRbWAcqGHsislcqaIXaqmaaaakeaacqWG4baEdaqhaaWcbaGaemyAaKMaeiilaWIaemyyaegabaGaem4AaSMaeyOeI0IaeGymaedaaaaaaeaacqWGPbqAcqGHiiIZcqWGvbqvdaWgaaadbaGaem4AaSgabeaaaSqab0Gaey4dIunaaOGaayjkaiaawMcaamaaCaaaleqabaGaeGymaeJaei4la8Iaei4iamYaaeWaaeaacqWGvbqvdaWgaaadbaGaem4AaSgabeaaaSGaayjkaiaawMcaaaaakiabcYcaSaaa@5A80@

or (the formula is equivalent)

pak=(∏i∈Ukxi,rk−1xi,a)1/#(Uk).
 MathType@MTEF@5@5@+=feaafiart1ev1aaatCvAUfKttLearuWrP9MDH5MBPbIqV92AaeXatLxBI9gBaebbnrfifHhDYfgasaacH8akY=wiFfYdH8Gipec8Eeeu0xXdbba9frFj0=OqFfea0dXdd9vqai=hGuQ8kuc9pgc9s8qqaq=dirpe0xb9q8qiLsFr0=vr0=vr0dc8meaabaqaciaacaGaaeqabaqabeGadaaakeaacqWGWbaCdaqhaaWcbaGaemyyaegabaGaem4AaSgaaOGaeyypa0ZaaeWaaeaadaqeqbqaamaalaaabaGaemiEaG3aa0baaSqaaiabdMgaPjabcYcaSiabdkhaYbqaaiabdUgaRjabgkHiTiabigdaXaaaaOqaaiabdIha4naaBaaaleaacqWGPbqAcqGGSaalcqWGHbqyaeqaaaaaaeaacqWGPbqAcqGHiiIZcqWGvbqvdaWgaaadbaGaem4AaSgabeaaaSqab0Gaey4dIunaaOGaayjkaiaawMcaamaaCaaaleqabaGaeGymaeJaei4la8Iaei4iamYaaeWaaeaacqWGvbqvdaWgaaadbaGaem4AaSgabeaaaSGaayjkaiaawMcaaaaakiabc6caUaaa@5120@

3. Update the signals using

xi,ak=pakxi,ak−1pak−1,
 MathType@MTEF@5@5@+=feaafiart1ev1aaatCvAUfKttLearuWrP9MDH5MBPbIqV92AaeXatLxBI9gBaebbnrfifHhDYfgasaacH8akY=wiFfYdH8Gipec8Eeeu0xXdbba9frFj0=OqFfea0dXdd9vqai=hGuQ8kuc9pgc9s8qqaq=dirpe0xb9q8qiLsFr0=vr0=vr0dc8meaabaqaciaacaGaaeqabaqabeGadaaakeaacqWG4baEdaqhaaWcbaGaemyAaKMaeiilaWIaemyyaegabaGaem4AaSgaaOGaeyypa0JaemiCaa3aa0baaSqaaiabdggaHbqaaiabdUgaRbaakmaalaaabaGaemiEaG3aa0baaSqaaiabdMgaPjabcYcaSiabdggaHbqaaiabdUgaRjabgkHiTiabigdaXaaaaOqaaiabdchaWnaaDaaaleaacqWGHbqyaeaacqWGRbWAcqGHsislcqaIXaqmaaaaaOGaeiilaWcaaa@481A@

or (again, equivalently)

xi,ak=pakxi,a.
 MathType@MTEF@5@5@+=feaafiart1ev1aaatCvAUfKttLearuWrP9MDH5MBPbIqV92AaeXatLxBI9gBaebbnrfifHhDYfgasaacH8akY=wiFfYdH8Gipec8Eeeu0xXdbba9frFj0=OqFfea0dXdd9vqai=hGuQ8kuc9pgc9s8qqaq=dirpe0xb9q8qiLsFr0=vr0=vr0dc8meaabaqaciaacaGaaeqabaqabeGadaaakeaacqWG4baEdaqhaaWcbaGaemyAaKMaeiilaWIaemyyaegabaGaem4AaSgaaOGaeyypa0JaemiCaa3aa0baaSqaaiabdggaHbqaaiabdUgaRbaakiabdIha4naaBaaaleaacqWGPbqAcqGGSaalcqWGHbqyaeqaaOGaeiOla4caaa@3EAA@

The algorithm has converged at step *m *if *U*_*m *_and *U*_*m*+1 _are identical sets. To start the algorithm at *k *= 1 we require initial values xi,a0
 MathType@MTEF@5@5@+=feaafiart1ev1aaatCvAUfKttLearuWrP9MDH5MBPbIqV92AaeXatLxBI9gBaebbnrfifHhDYfgasaacH8akY=wiFfYdH8Gipec8Eeeu0xXdbba9frFj0=OqFfea0dXdd9vqai=hGuQ8kuc9pgc9s8qqaq=dirpe0xb9q8qiLsFr0=vr0=vr0dc8meaabaqaciaacaGaaeqabaqabeGadaaakeaacqWG4baEdaqhaaWcbaGaemyAaKMaeiilaWIaemyyaegabaGaeGimaadaaaaa@32C6@. These are given by

xi,a0
 MathType@MTEF@5@5@+=feaafiart1ev1aaatCvAUfKttLearuWrP9MDH5MBPbIqV92AaeXatLxBI9gBaebbnrfifHhDYfgasaacH8akY=wiFfYdH8Gipec8Eeeu0xXdbba9frFj0=OqFfea0dXdd9vqai=hGuQ8kuc9pgc9s8qqaq=dirpe0xb9q8qiLsFr0=vr0=vr0dc8meaabaqaciaacaGaaeqabaqabeGadaaakeaacqWG4baEdaqhaaWcbaGaemyAaKMaeiilaWIaemyyaegabaGaeGimaadaaaaa@32C6@ = pa0
 MathType@MTEF@5@5@+=feaafiart1ev1aaatCvAUfKttLearuWrP9MDH5MBPbIqV92AaeXatLxBI9gBaebbnrfifHhDYfgasaacH8akY=wiFfYdH8Gipec8Eeeu0xXdbba9frFj0=OqFfea0dXdd9vqai=hGuQ8kuc9pgc9s8qqaq=dirpe0xb9q8qiLsFr0=vr0=vr0dc8meaabaqaciaacaGaaeqabaqabeGadaaakeaacqWGWbaCdaqhaaWcbaGaemyyaegabaGaeGimaadaaaaa@307B@*x_i, a_*,     (7)

where we will typically fix pa0
 MathType@MTEF@5@5@+=feaafiart1ev1aaatCvAUfKttLearuWrP9MDH5MBPbIqV92AaeXatLxBI9gBaebbnrfifHhDYfgasaacH8akY=wiFfYdH8Gipec8Eeeu0xXdbba9frFj0=OqFfea0dXdd9vqai=hGuQ8kuc9pgc9s8qqaq=dirpe0xb9q8qiLsFr0=vr0=vr0dc8meaabaqaciaacaGaaeqabaqabeGadaaakeaacqWGWbaCdaqhaaWcbaGaemyyaegabaGaeGimaadaaaaa@307B@ = 1.

Several criteria have to be met for the algorithm to be reliable. First, one has to verify that the algorithm does converge: it has to find the final set *U*_*m *_and not wander aimlessly among the huge number of gene sets. Second, the convergence has to be reasonably quick and robust. Lastly, one wants to ensure that the final set of genes *U*_*m *_returned by the algorithm is indeed a set of constant genes. These conditions are difficult to verify analytically, which is why we resorted to numeric simulation. These simulations are presented in the Results and Methods section. A R script implementing the algorithm in the case of simple replication and no extra conditions has been included as supplementary material.

### A set of constant genes can be used for rescaling

We want here to prove that (3) holds and in particular demonstrate that the approximation is a good one. Using (1) we have

p˜a=krka(∏i∈Usi)1/#(U)(∏i∈Usi)1/#(U)(∏i∈Uexp⁡(εi,r))1/#(U)(∏i∈Uexp⁡(εi,a))1/#(U).     (8)
MathType@MTEF@5@5@+=feaafiart1ev1aaatCvAUfKttLearuWrP9MDH5MBPbIqV92AaeXatLxBI9gBaebbnrfifHhDYfgasaacH8akY=wiFfYdH8Gipec8Eeeu0xXdbba9frFj0=OqFfea0dXdd9vqai=hGuQ8kuc9pgc9s8qqaq=dirpe0xb9q8qiLsFr0=vr0=vr0dc8meaabaqaciaacaGaaeqabaqabeGadaaakeaacuWGWbaCgaacamaaBaaaleaacqWGHbqyaeqaaOGaeyypa0ZaaSaaaeaacqWGRbWAdaWgaaWcbaGaemOCaihabeaaaOqaaiabdUgaRnaaBaaaleaacqWGHbqyaeqaaaaakmaalaaabaWaaeWaaeaadaqeqaqaaiabdohaZnaaBaaaleaacqWGPbqAaeqaaaqaaiabdMgaPjabgIGiolabdwfavbqab0Gaey4dIunaaOGaayjkaiaawMcaamaaCaaaleqabaGaeGymaeJaei4la8Iaei4iamYaaeWaaeaacqWGvbqvaiaawIcacaGLPaaaaaaakeaadaqadaqaamaarababaGaem4Cam3aaSbaaSqaaiabdMgaPbqabaaabaGaemyAaKMaeyicI4SaemyvaufabeqdcqGHpis1aaGccaGLOaGaayzkaaWaaWbaaSqabeaacqaIXaqmcqGGVaWlcqGGJaWidaqadaqaaiabdwfavbGaayjkaiaawMcaaaaaaaGcdaWcaaqaamaabmaabaWaaebeaeaacyGGLbqzcqGG4baEcqGGWbaCdaqadaqaaGGaciab=v7aLnaaBaaaleaacqWGPbqAcqGGSaalcqWGYbGCaeqaaaGccaGLOaGaayzkaaaaleaacqWGPbqAcqGHiiIZcqWGvbqvaeqaniabg+GivdaakiaawIcacaGLPaaadaahaaWcbeqaaiabigdaXiabc+caViabcocaJmaabmaabaGaemyvaufacaGLOaGaayzkaaaaaaGcbaWaaeWaaeaadaqeqaqaaiGbcwgaLjabcIha4jabcchaWnaabmaabaGae8xTdu2aaSbaaSqaaiabdMgaPjabcYcaSiabdggaHbqabaaakiaawIcacaGLPaaaaSqaaiabdMgaPjabgIGiolabdwfavbqab0Gaey4dIunaaOGaayjkaiaawMcaamaaCaaaleqabaGaeGymaeJaei4la8Iaei4iamYaaeWaaeaacqWGvbqvaiaawIcacaGLPaaaaaaaaOGaeiOla4IaaCzcaiaaxMaadaqadaqaaiabiIda4aGaayjkaiaawMcaaaaa@8BDE@

We have *k*_*r *_= 1, the second ratio on the right hand side is equal to 1. Turning to the third term in (8), we see that its expectation can be written as

*E *(exp (*ε*)),

where

ε=1#(U)∑i∈Uεi,r−1#(U)∑i∈Uεi,a.     (9)
 MathType@MTEF@5@5@+=feaafiart1ev1aaatCvAUfKttLearuWrP9MDH5MBPbIqV92AaeXatLxBI9gBaebbnrfifHhDYfgasaacH8akY=wiFfYdH8Gipec8Eeeu0xXdbba9frFj0=OqFfea0dXdd9vqai=hGuQ8kuc9pgc9s8qqaq=dirpe0xb9q8qiLsFr0=vr0=vr0dc8meaabaqaciaacaGaaeqabaqabeGadaaakeaaiiGacqWF1oqzcqGH9aqpdaWcaaqaaiabigdaXaqaaiabcocaJmaabmaabaGaemyvaufacaGLOaGaayzkaaaaamaaqafabaGae8xTdu2aaSbaaSqaaiabdMgaPjabcYcaSiabdkhaYbqabaaabaGaemyAaKMaeyicI4SaemyvaufabeqdcqGHris5aOGaeyOeI0YaaSaaaeaacqaIXaqmaeaacqGGJaWidaqadaqaaiabdwfavbGaayjkaiaawMcaaaaadaaeqbqaaiab=v7aLnaaBaaaleaacqWGPbqAcqGGSaalcqWGHbqyaeqaaaqaaiabdMgaPjabgIGiolabdwfavbqab0GaeyyeIuoakiabc6caUiaaxMaacaWLjaWaaeWaaeaacqaI5aqoaiaawIcacaGLPaaaaaa@5544@

For the sake of this argument, we assume the relative errors for the gene expression measurements to be iid and equal to 20%. Applying the central limit theorem to (9), one can see that *ε *is Gaussian with parameters *μ *= 0 and *a *= (2 * 0.2)/#(U)
 MathType@MTEF@5@5@+=feaafiart1ev1aaatCvAUfKttLearuWrP9MDH5MBPbIqV92AaeXatLxBI9gBaebbnrfifHhDYfgasaacH8akY=wiFfYdH8Gipec8Eeeu0xXdbba9frFj0=OqFfea0dXdd9vqai=hGuQ8kuc9pgc9s8qqaq=dirpe0xb9q8qiLsFr0=vr0=vr0dc8meaabaqaciaacaGaaeqabaqabeGadaaakeaadaGcaaqaaiabcocaJmaabmaabaGaemyvaufacaGLOaGaayzkaaaaleqaaaaa@3051@. Thus, exp(*ε*) is lognormal with the same parameters. The expectation of a lognormal distribution is exp(*μ *+ *σ*^2^/2), hence

*E *(exp (*ε*)) = exp(0 + 0.08/#(*U*)).

The expectation of this third term is slightly bigger than 1 but since the size on the set *U *is typically in the hundreds, the difference is considerably less than 1%. Replacing in (8) we thus have, as required

p˜a≈1ka=pa.
 MathType@MTEF@5@5@+=feaafiart1ev1aaatCvAUfKttLearuWrP9MDH5MBPbIqV92AaeXatLxBI9gBaebbnrfifHhDYfgasaacH8akY=wiFfYdH8Gipec8Eeeu0xXdbba9frFj0=OqFfea0dXdd9vqai=hGuQ8kuc9pgc9s8qqaq=dirpe0xb9q8qiLsFr0=vr0=vr0dc8meaabaqaciaacaGaaeqabaqabeGadaaakeaacuWGWbaCgaacamaaBaaaleaacqWGHbqyaeqaaOGaeyisIS7aaSaaaeaacqaIXaqmaeaacqWGRbWAdaWgaaWcbaGaemyyaegabeaaaaGccqGH9aqpcqWGWbaCdaWgaaWcbaGaemyyaegabeaakiabc6caUaaa@3A0A@

where the approximation is more than good enough for our practical purposes.

### In silico explorations

#### Artificial data generation

We generated artificial signals for 10,000 genes on 7 microarrays using the following procedure. For the *i*th gene in the *a*th microarray we generate observed signals *x*_*i*,*a *_according to

*x*_*i*,*a *_= *k*_*a *_*s*_*i*,*a *_exp(*ε*),

The first term *k*_*a *_is the scaling mismatch we want the algorithm to uncover. The third term *exp*(*ε*) is an error term, where *ε *is set to be Gaussian with mean zero and standard deviation 0.2. The second term *s*_*i*,*a *_is generated using

si,a=10G,
 MathType@MTEF@5@5@+=feaafiart1ev1aaatCvAUfKttLearuWrP9MDH5MBPbIqV92AaeXatLxBI9gBamrtHrhAL1wy0L2yHvtyaeHbnfgDOvwBHrxAJfwnaebbnrfifHhDYfgasaacH8akY=wiFfYdH8Gipec8Eeeu0xXdbba9frFj0=OqFfea0dXdd9vqai=hGuQ8kuc9pgc9s8qqaq=dirpe0xb9q8qiLsFr0=vr0=vr0dc8meaabaqaciaacaGaaeqabaWaaeGaeaaakeaacqWGZbWCdaWgaaWcbaGaemyAaKMaeiilaWIaemyyaegabeaakiabg2da9iabigdaXiabicdaWmaaCaaaleqabaacdaGae8NbXFeaaOGaeiilaWcaaa@4153@

where G
 MathType@MTEF@5@5@+=feaafiart1ev1aaatCvAUfKttLearuWrP9MDH5MBPbIqV92AaeXatLxBI9gBamrtHrhAL1wy0L2yHvtyaeHbnfgDOvwBHrxAJfwnaebbnrfifHhDYfgasaacH8akY=wiFfYdH8Gipec8Eeeu0xXdbba9frFj0=OqFfea0dXdd9vqai=hGuQ8kuc9pgc9s8qqaq=dirpe0xb9q8qiLsFr0=vr0=vr0dc8meaabaqaciaacaGaaeqabaWaaeGaeaaakeaaimaacqWFge=raaa@382D@ is a Gaussian random variable with mean 1.5 and standard deviation 0.4. Furthermore, a small portion of these genes (5% or 500) are set to be constant, that is for *i ε U*, we have *s*_*i*,*a *_= *s*_*i*_.

Alhough real experiments yield more complex expression patterns, this artificial data is amply sufficient to capture the main features of our method.

To simulate the scaling mismatches *k*_*a*_, we generate them by

*k*_*a *_= 1 + *K*(*u *- 0.5),     (10)

where *u *is a random variable uniformly distributed on the unit interval and *K *is a parameter capturing the severity of the mismatch. Without loss of generality, we set *k*_1 _= 1 because choosing an arbitrary reference array is required by our method.

We generated 191 different datasets and ran the algorithm on each of them. Each dataset differed from the others by the choice of the constant *K *in (10). The 191 values for *K *ranged uniformly between 0 and 1.9.

Each run was then assigned to one of the following three groups on the basis of the size of the final set *U*_*m *_(where the index *m *denotes the number of steps it took to the algorithm to converge):

• *Good *runs were those for which the size of the final set *U*_*m *_exceeded 100.

• In contrast, *poor *runs were those for which the size of *U*_*m *_was less than or equal to 100.

• In the special case where the algorithm failed to find a big enough (> 2) *initial *set *U*_1_, three genes known to be constant were forced into the initial set (recall that 5% of the genes were set to be constant). We call these *helped *runs.

For each run *r*, we computed an accuracy index using

accuracy=16∑a=27|1−paka|     (11)
 MathType@MTEF@5@5@+=feaafiart1ev1aaatCvAUfKttLearuWrP9MDH5MBPbIqV92AaeXatLxBI9gBaebbnrfifHhDYfgasaacH8akY=wiFfYdH8Gipec8Eeeu0xXdbba9frFj0=OqFfea0dXdd9vqai=hGuQ8kuc9pgc9s8qqaq=dirpe0xb9q8qiLsFr0=vr0=vr0dc8meaabaqaciaacaGaaeqabaqabeGadaaakeaacqWGHbqycqWGJbWycqWGJbWycqWG1bqDcqWGYbGCcqWGHbqycqWGJbWycqWG5bqEcqGH9aqpdaWcaaqaaiabigdaXaqaaiabiAda2aaadaaeWbqaamaaemaabaGaeGymaeJaeyOeI0IaemiCaa3aaSbaaSqaaiabdggaHbqabaGccqWGRbWAdaWgaaWcbaGaemyyaegabeaaaOGaay5bSlaawIa7aaWcbaGaemyyaeMaeyypa0JaeGOmaidabaGaeG4naCdaniabggHiLdGccaWLjaGaaCzcamaabmaabaGaeGymaeJaeGymaedacaGLOaGaayzkaaaaaa@508B@

where *p*_*a *_is the rescaling factor recovered from thealgorithm. Ideally, we want the rescaling factors *p*_*a *_to perfectly compensate the scaling factors *k*_*a *_so that *p*_*a*_*k*_*a *_= 1. Consequently, the accuracy index above, which can take non-negative values, must be as close to zero as possible. We also monitored the speed of convergence i.e. the number of iterations steps *m *the algorithm takes before convergence.

## Authors' contributions

MB designed the method in collaboration with JS and wrote the paper. DT and MH ran the microarrays. RC and DB provided useful advice. MH, JS and RC designed and coordinated the project.

## Availability

An R script implementing the algorithm in the case of simple replication and no extra conditions has been included as additional file 1 (rescale.R). The data is available in Array Express (accession number E-MEXP-549).

## Supplementary Material

Additional File 1 An R script implementing the algorithm in the case of simple replication and no extra conditions has been included as additional file (rescale.R). Click here for file

## Figures and Tables

**Figure 1:**
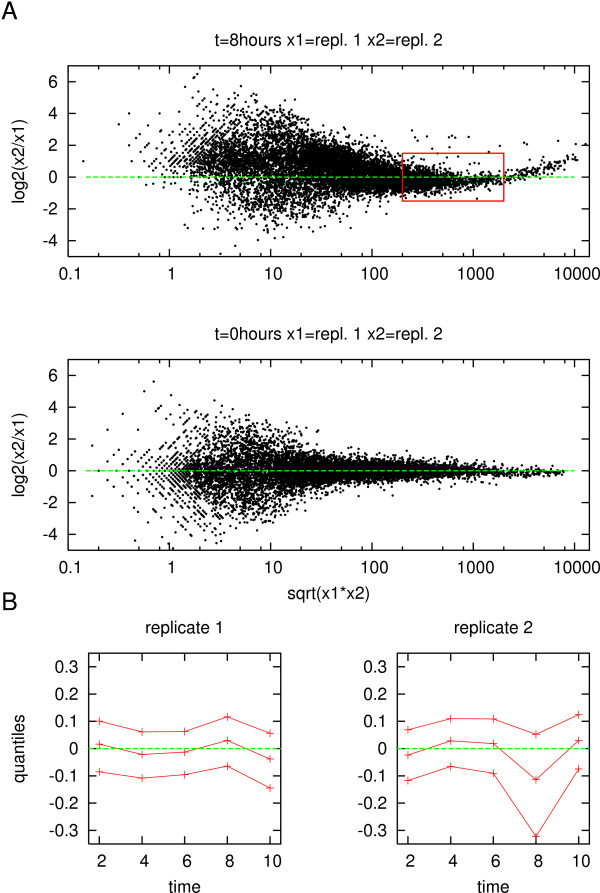
**Effect of different transcript concentrations illustrated by MvA plots**. A) An MvA plots of mis-scaled data (top) and normal data (bottom). In the top plot, the combined effect of non-linear signal response and differences in transcript concentration causes the points to lie in curved region (replicate 2 is the darker microarray, both replicates taken 8 hours after irradiation). In contrast, when transcript concentrations are similar the cloud of points is not curved, but aligned with the horizontal axis (bottom plot, replicates 1 and 2 for the 0 hours time point). Focusing back to the top plot (region boxed in red), one can see how scaling mismatches occur in the median signal range: since the scaling performed in MAS5 takes into account almost all the signals, the signals of those genes lying in the median signal range are underscaled on the second chip: the median section of the cloud is underneath the horizontal axis. B) The scaling mismatch is even more striking when considering a simple time course application using the raw data. Within each replicate, intermediate time points have been linearly interpolated using neighbouring time points (e.g. for the 2 hours time point, the 0 and 4 hours chips have been used). The relative difference between those interpolation results and the actual values have been computed and the graphs show, for each intermediate time point, the first, second, and third quartile of the distribution of these relative differences. The scaling imbalance that occur with non-rescaled data is particularly visible for the 8 hours time point on the second replicate. To see how our algorithm corrects this problem, see Figure 6.

**Figure 2:**
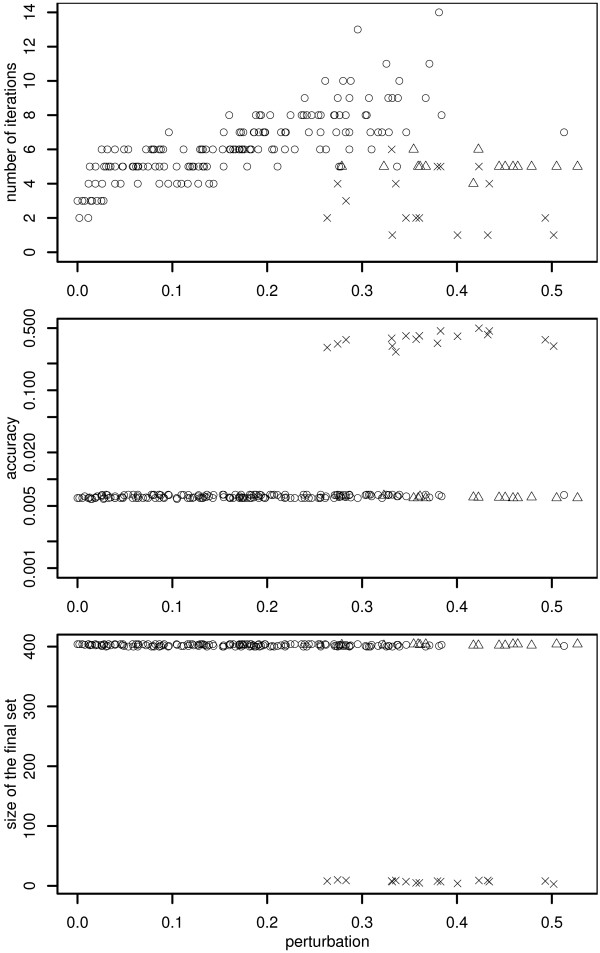
**Algorithm performance for a range of perturbations, with 5% of constant genes**. This series of three plots illustrates the performance of the algorithm. Each run of the algorithm is represented by a symbol in all three plots. The symbol "○" represents good runs, "×" represents poor runs and "△" represents helped runs. In all three plots, the horizontal axis gives the *effective *perturbation (the average of the absolute difference to 1 of all scaling mismatches *k*_*a*_). The vertical axis in the top plot is the number of iterations required to reach convergence. In the second plot, we present the accuracy of the rescaling factors (11). In the bottom plot, the vertical axis gives the size of the final set *U*_*m*_. For all the runs represented in those plots, 5% of the genes were pre-set to be constant.

**Figure 3:**
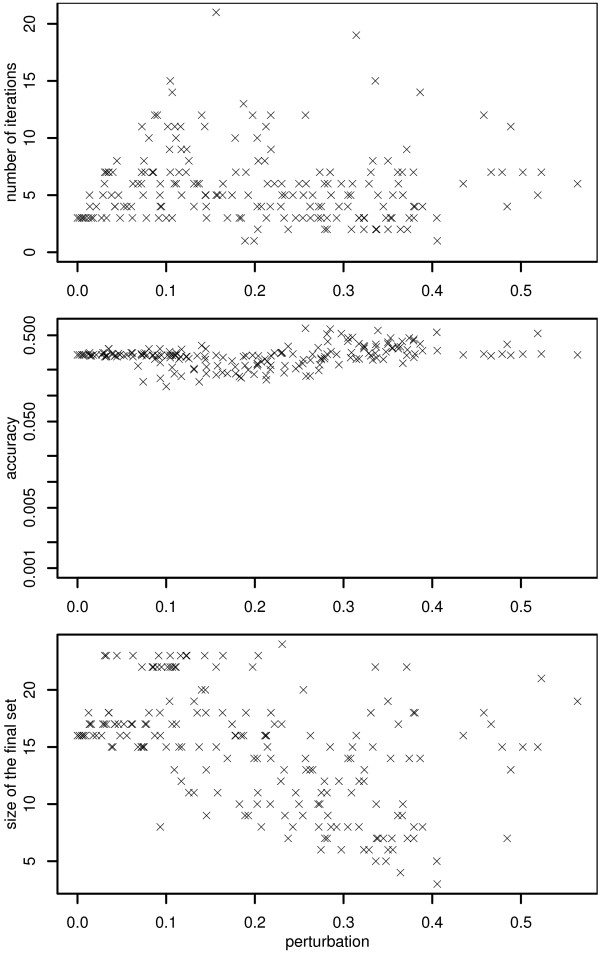
**Algorithm performance for a range of perturbation, with no constant genes**. This series of three plots illustrates the performance of the algorithm. Each run of the algorithm is represented by a symbol in all three plots. The symbol "×" represents poor runs. In all three plots, the horizontal axis gives the *effective *perturbation (the average of the absolute difference to 1 of all scaling mismatches *k*_*a*_). The vertical axis in the top plot is the number of iterations required to reach convergence. In the second plot, we present the accuracy of the rescaling factors (11). In the bottom plot, the vertical axis gives the size of the final set *U*_*m*_. This series of plots are obtained through the same process shown in Figure 2 except that no genes were forced to be constant. In this situation, the algorithm yields only poor results, there are no good runs.

**Figure 4:**
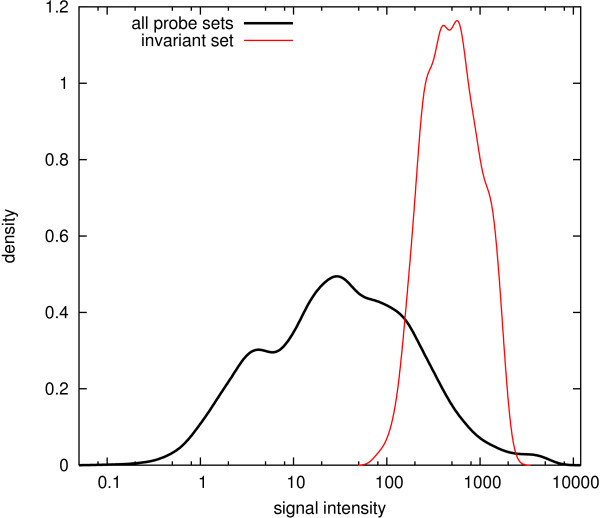
**Signal distribution for the set of constant genes, compared to the overall signal distribution**. This plot compares the density of the overall distribution of expression in the microarray (black) to the one for the constant genes set (red). As intended, the distribution range of the constant genes set is narrower and centred on the signal range of interest. Both density curves are for the second time point (2 hours after irradiation) of the first replicate.

**Figure 5:**
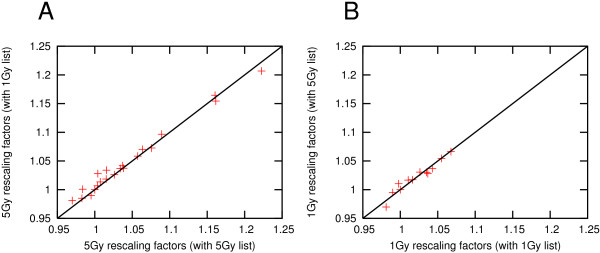
**Rescaling factors obtained with different lists**. This figure represents the rescaling factors for both sets of microarrays each cross represents a microarray. Both 5Gy (A) and 1Gy (B) are shown. The horizontal coordinate gives the rescaling factor obtained with the original list and the rescaling factor extracted from the other experiment is given by the vertical coordinate. Both sets of rescaling factors are very similar showing that lists of constant genes in one experiment can be re-used in another one, provided that the tissues and experimental conditions used are similar.

**Figure 6:**
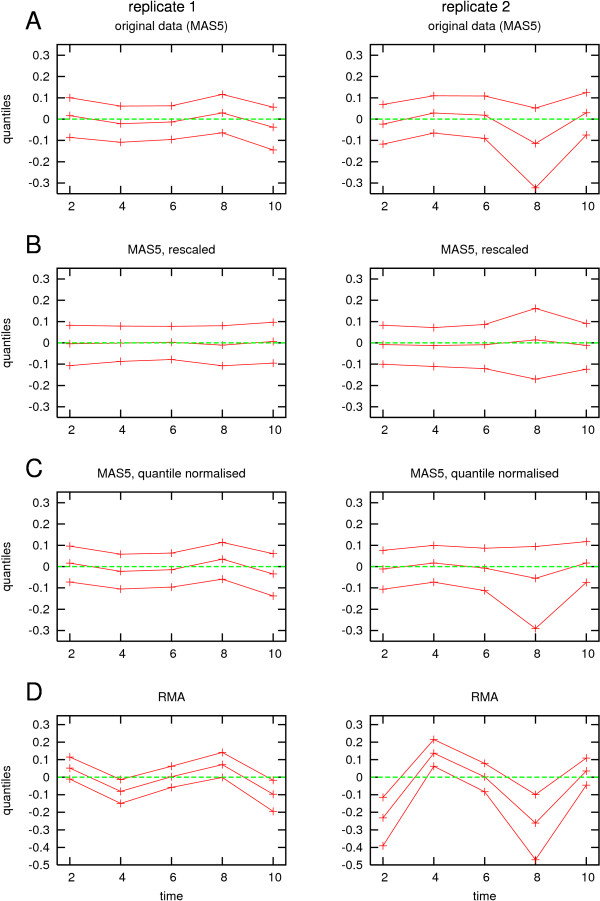
**Effects of rescaling, quantile normalization and a different summary procedure on subsequent analysis**. Intermediate time points were interpolated using a simple linear procedure using neighbouring time points (for example, the 8 hours time point was interpolated using the 6 and 10 hours time points). A relative interpolation error was then computed from each gene using the formula (*X_T _- X_P_*)/*X_T _*where *X_T _*is the true value and *X_P _*the predicted one. In each plot there are three curves which, from top to bottom correspond to the third quartile, median and first quartile of the relative interpolation error. This procedure was repeated for each replicate separately before (A), after (B) rescaling, after quantile normalization applied on MAS5 summarized values (C) and on RMA summarized data (D). In (B) scaling imbalances have been corrected whilst the performance of quantile normalization and RMA summarized data are inferior in that respect (C, D). Only those genes whose neighbouring time points have a detection p-value less than or equal to 0.001 were included, which corresponds to 2, 500 – 5, 000 genes/time point.

**Table 1:** Rescaling factors obtained by the recursive procedure (5Gy experiment)

	sample 1	sample 2	sample 3
0 hrs	1.00	0.99	0.97
2 hrs	0.98	1.02	1.00
4 hrs	1.04	1.01	0.98
6 hrs	1.04	1.09	1.02
8 hrs	1.00	1.22	1.16
10 hrs	1.08	1.06	1.16
12 hrs	1.03	1.03	1.06

**Table 2:** Original scaling factors obtained with MAS5

	sample 1	sample 2	sample 3
0 hrs	0.852	0.751	0.931
2 hrs	0.667	0.949	1.925
4 hrs	0.675	1.233	1.019
6 hrs	0.882	1.797	1.732
8 hrs	0.744	3.010	2.634
10 hrs	1.121	1.232	2.364
12 hrs	1.066	1.134	1.688

**Table 3:** Rescaling factors obtained by the recursive procedure (1Gy experiment)

	Sample 1	Sample 2	Sample 3
0 hrs	1.00	0.99	0.98
4 hrs	1.00	1.03	1.02
8 hrs	1.03	1.07	1.04
12 hrs	1.01	1.04	1.06
